# *Leishmania*-Mediated Inhibition of Iron Export Promotes Parasite Replication in Macrophages

**DOI:** 10.1371/journal.ppat.1003901

**Published:** 2014-01-30

**Authors:** Rym Ben-Othman, Andrew R. Flannery, Danilo C. Miguel, Diane M. Ward, Jerry Kaplan, Norma W. Andrews

**Affiliations:** 1 Department of Cell Biology and Molecular Genetics, University of Maryland, College Park, Maryland, United States of America; 2 Department of Pathology, University of Utah, Salt Lake City, Utah, United States of America; Jawaharlal Nehru University, India

## Abstract

*Leishmania* parasites infect macrophages, cells that play an important role in organismal iron homeostasis. By expressing ferroportin, a membrane protein specialized in iron export, macrophages release iron stored intracellularly into the circulation. Iron is essential for the intracellular replication of *Leishmania*, but how the parasites compete with the iron export function of their host cell is unknown. Here, we show that infection with *Leishmania amazonensis* inhibits ferroportin expression in macrophages. In a TLR4-dependent manner, infected macrophages upregulated transcription of hepcidin, a peptide hormone that triggers ferroportin degradation. Parasite replication was inhibited in hepcidin-deficient macrophages and in wild type macrophages overexpressing mutant ferroportin that is resistant to hepcidin-induced degradation. Conversely, intracellular growth was enhanced by exogenously added hepcidin, or by expression of dominant-negative ferroportin. Importantly, dominant-negative ferroportin and macrophages from flatiron mice, a mouse model for human type IV hereditary hemochromatosis, restored the infectivity of mutant parasite strains defective in iron acquisition. Thus, inhibition of ferroportin expression is a specific strategy used by *L. amazonensis* to inhibit iron export and promote their own intracellular growth.

## Author Summary

Infection with the protozoan parasite *Leishmania* causes significant human disease in many parts of the world, particularly in the Middle East, India and South America. The parasite is transmitted by sand flies, which are difficult to control and are becoming increasingly common in urban areas. With domestic dogs serving as reservoirs of the disease and global travel increasing the population of infected human patients, the overall burden of leishmaniasis is on the rise. In mammals these parasites replicate inside macrophages, and therefore need strategies to survive within a cell that is specialized in killing pathogens. Earlier work demonstrated that iron is one of the essential nutrients that *Leishmania* must acquire from host cells to survive. Acquiring iron is particularly challenging inside macrophages, which play an important role in host iron homeostasis and export iron extracellularly through the membrane transporter ferroportin. We found that *Leishmania amazonesis* induces their host macrophages to produce hepcidin, a peptide that triggers internalization and degradation of ferroportin. This strategy increases the macrophage intracellular iron pool, and stimulates *Leishmania* replication. These results suggest that defects in iron homeostasis, which occur frequently in the human population, can have an important role in susceptibility to *Leishmania* infections.

## Introduction

Intracellular parasites must obtain essential nutrients from their host cells. Iron is a vital nutritional resource for mammalian hosts and also for many pathogens, acting as an essential cofactor of proteins and enzymes involved in metabolic pathways. Under physiological conditions, iron is normally found in the insoluble, oxidized Fe^3+^ form associated with carrier proteins such as transferrin and ferritin. After endocytosis mediated by transferrin receptors, Fe^3+^ is reduced to the soluble Fe^2+^ form and translocated to the cytosol. In the cytosol, iron may be utilized by the host, stored as complex with ferritin, or exported out of the cells. Thus, the pool of available iron within mammalian cells is determined by a carefully orchestrated balance between uptake through plasma membrane and endosomal receptors/transporters and cellular export.

The only known mammalian iron exporter is ferroportin (Fpn1), a protein with an essential role in iron homeostasis [1]. Fpn1 is expressed on the surface of cells specialized in storage or transport of iron, including enterocytes, hepatocytes and macrophages. Transcription of *Fpn1* is increased by several factors, including iron. Further, *Fpn1* mRNA has a 5′iron-responsive element (IRE) that limits its translation when iron availability is low and enhances it under high iron conditions. Fpn1 levels on the cell surface are regulated by hepcidin, a peptide hormone that triggers Fpn1 internalization and lysosomal degradation [2]. Modulation of Fpn1 expression allows macrophages to function as mediators of iron homeostasis in vivo, responding to systemic and localized signals by retaining or exporting iron [3], [4]. Missense mutations in *Fpn1* are autosomal dominant in humans and some mutations lead to hereditary hemochromatosis type IV (form A), a human disease characterized by high ferritin levels, low transferrin saturation and iron accumulation inside macrophages [5]. Other *Fpn1* mutations lead to constitutive iron export due to the inability to respond to hepcidin.

The protozoan parasite *Leishmania*, which causes serious infections in millions of people throughout the world, replicates mainly in macrophages. Until recently very little was known about mechanisms of iron acquisition and utilization by *Leishmania*, and how changes in host cell iron homeostasis affect these intracellular parasites. Recent studies improved this scenario, by identifying three *L. amazonensis* membrane proteins that mediate iron uptake and are required for parasite virulence: the Fe^3+^ reductase LFR1 [6], the Fe^2+^ transporter LIT1 [7] and the heme transporter LHR1 [8]. In *L. donovani*, the ATP-binding cassette membrane protein LABCG5 was also implicated in the salvage of heme generated by degradation of hemoglobin in parasite lysosomes [9]. These studies showed that the iron-poor environment of macrophage phagolysosomes induces *Leishmania* to express molecules that promote uptake of iron and heme, factors essential for intracellular survival and replication. However, it is still not understood how the parasites compete with the highly developed pathways of iron export and storage of macrophages to gain access to iron. In this study we examined how iron efflux and modulation of host cell iron content affect the intracellular growth of *L. amazonensis*. Our results revealed a parasite strategy for inhibition of iron export that is required for the establishment of productive infections in macrophages.

## Results

### Infection with *L. amazonensis* inhibits macrophage expression of Fpn1 in a TLR4-dependent manner

**Figure 1:**
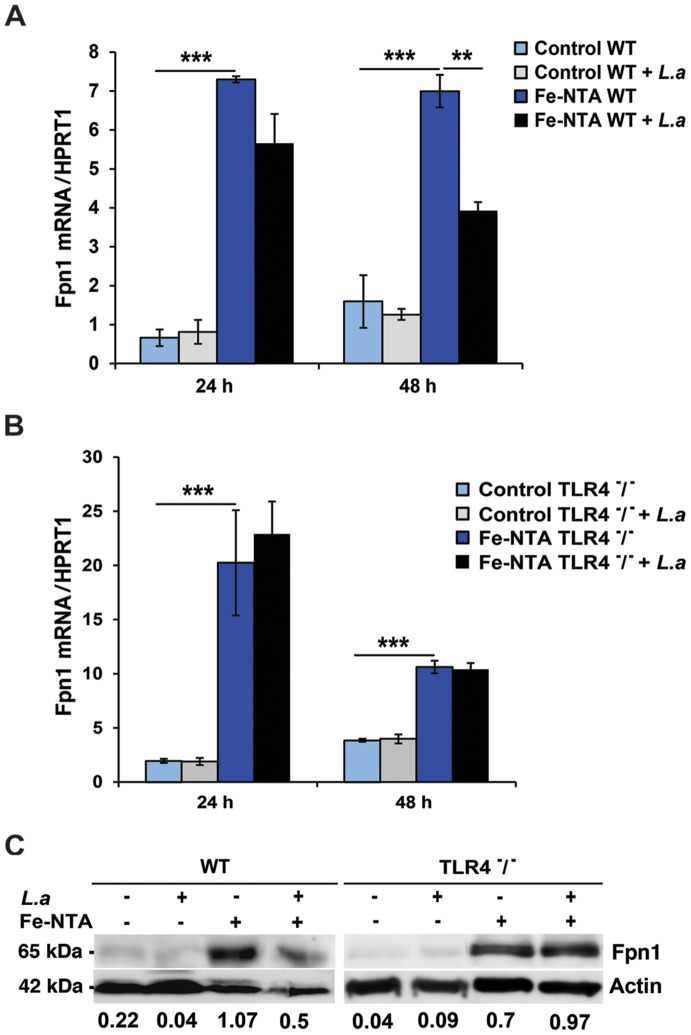
*L. amazonensis* infection inhibits Fpn1 expression in macrophages in a TLR4 dependent manner. BMDM were left untreated (Control) or incubated with Fe-NTA overnight, washed and infected or not with *L. amazonensis* axenic amastigotes (*L.a*) for 1 h. BMDM were washed to remove extracellular parasites, cultured at 34°C for another 24 or 48 h and *Fpn1* or hypoxantine phosphoribosyltransferase (*HPRT1*) mRNA was quantified by qPCR. (**A**) Effect of *L. amazonensis* infection on ferroportin (*Fpn1*) mRNA in wild type (WT) BMDM. (**B**) Effect of *L. amazonensis* infection on ferroportin (*Fpn1*) mRNA in TLR4^−/−^ BMDM. The values represent *Fpn1* mRNA levels normalized to *HRPT1* (mean +/− SD of triplicates). (**) p<0.01; (***) p<0.001 (unpaired Student's t test). (**C**) Fpn1 protein levels in WT and TLR4^−^/^−^ BMDM treated as in (A, B) and infected or not with *L. amazonensis* axenic amastigotes (*L.a*) for 48 h. BMDM lysates were analysed by Western Blot with anti-Fpn1 antibodies. Anti-actin antibodies were used as loading controls. Fpn1/actin ratios were determined by quantitative digital imaging. The data are representative of at least 2 independent experiments.

We investigated the impact of infection with *L. amazonensis* on the expression of Fpn1 in mouse bone marrow-derived macrophages (BMDM). Low levels of *Fpn1* transcripts were detected in wild type and TLR4^−/−^ BMDM with and without *L. amazonensis* infection (Figure 1A,B). In agreement with previous studies showing *Fpn1* upregulation in response to iron loading [10], [11], treatment with Fe-nitrilotriacetate (Fe-NTA) for 16 h markedly increased *Fpn1* transcripts (Figure 1A,B). Following infection with *L. amazonensis*, *Fpn1* mRNA levels were reduced in Fe-NTA-treated wild type BMDM (Figure 1A). In contrast, although TLR4^−/−^ BMDM showed higher amounts of *Fpn1* mRNA (particularly 24 h after Fe-NTA treatment), the transcript levels were very similar between infected and non-infected cells (Figure 1B). At the protein level, immunoblots detected low amounts of Fpn1 in untreated BMDM and elevated levels 48 h after Fe-NTA treatment, in non-infected BMDM derived from either wild type or TLR4^−/−^ mice (Figure 1C). Similar to what was observed with the mRNA, infection with *L. amazonensis* reduced the amounts of Fpn1 protein in Fe-NTA-treated wild type, but not TLR4^−/−^ BMDM. Fpn1 levels detected in BMDM not treated with Fe-NTA were low, but quantification and ratio determinations in relation to actin showed a similar trend under these conditions as well - Fpn1was reduced in infected wild type, but not in infected TLR4^−/−^ BMDM (Figure 1C). Similar experiments performed with BMDM from TLR2^−/−^ mice showed results undistinguishable from wild type (data not shown). These findings suggest that the signalling pathway involved in *Fpn1* downregulation by *L. amazonensis* is TLR4-dependent.

### *L. amazonensis* infection induces TLR4-dependent expression of hepcidin in macrophages

**Figure 2:**
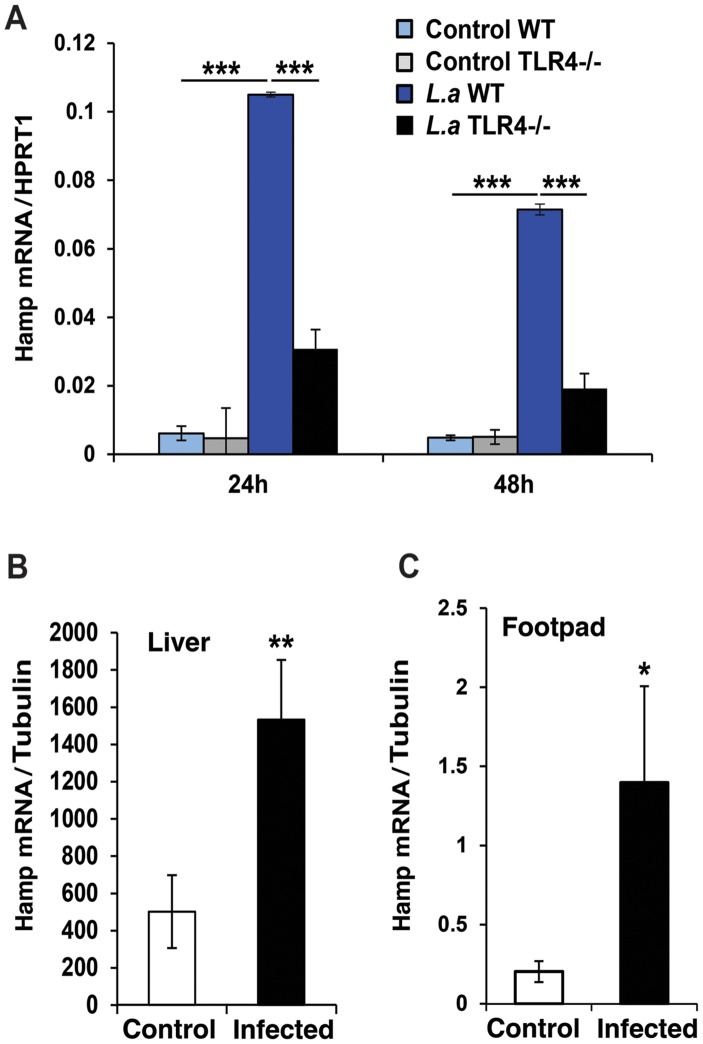
*L. amazonensis* infection enhances hepcidin expression in macrophages in a TLR4 dependent manner. WT and TLR4^−^/^−^ BMDM were infected or not with *L. amazonensis* axenic amastigotes (*L.a*) for 1 h and *Hamp* mRNA was quantified by qPCR. (**A**) Effect of *L. amazonensis* infection on hepcidin (*Hamp*) transcript levels. The values represent *Hamp* mRNA levels normalized to *HRPT1* (mean +/− SD of triplicates). The data are representative of at least 3 independent experiments. (***) p<0.001 (unpaired Student's t test). (**B,C**) Livers and footpads from uninfected or 9-week infected mice were homogenized and *Hamp* transcripts were quantified by real time PCR. The values represent *Hamp* mRNA levels normalized to tubulin (mean +/− SD of triplicates). The values represent the mean +/− SD of assays performed with tissues of 3 independent mice. (*) p<0.05; (**) p<0.01 (unpaired Student's t test).

The peptide hormone hepcidin is a potent regulator of iron export in macrophages, inducing Fpn1 internalization and degradation [2], [12]. We found that *L. amazonensis* infection upregulates hepcidin (*Hamp*) transcripts in BMDM (Figure 2A), an effect not observed with heat-killed parasites (data not shown). The ability of *L. amazonensis* to induce hepcidin expression was TLR4-dependent, consistent with what was observed for Fpn1 downregulation (Figure 1C). Importantly, upregulation of *Hamp* transcripts was also observed *in vivo*, in the liver and in footpad tissue associated with cutaneous lesions of mice infected for 9 weeks with *L. amazonensis* (Figure 2B,C) (see also supporting information S1). These results strongly suggest that macrophages infected with *L. amazonensis* express hepcidin.

### *L. amazonensis* infection induces iron accumulation and ferritin upregulation in macrophages

**Figure 3:**
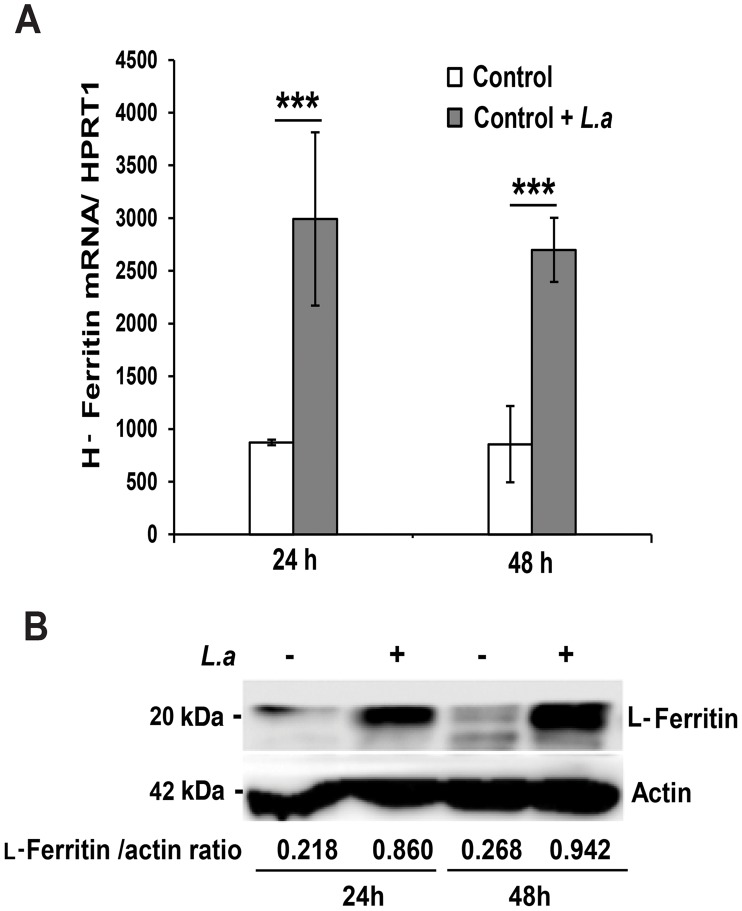
*L. amazonensis* infection causes iron accumulation and ferritin upregulation in macrophages. (**A**) Effect of *L. amazonensis* infection on H-ferritin (*FTH1*) transcript levels. BMDM were infected or not with axenic *L. amazonensis* amastigotes (*L.a*) for 1 h, washed and cultured at 34°C for another 24 or 48 h, followed by quantification of *FTH1* and *HPRT1* mRNA by qPCR. The values represent *FTH1* mRNA levels normalized to *HRPT1* (mean +/− SD of triplicates). (***) p<0.001 (unpaired Student's t test). (**B**) Effect of *L. amazonensis* infection on L-ferritin protein levels. BMDM infected or not with *L. amazonensis* axenic amastigotes (*L.a*) were solubilized after 24 or 48 h and analysed by Western Blot with anti-L-ferritin antibodies. Anti-actin antibodies were used as loading controls. L-ferritin/actin ratios were determined by quantitative digital imaging. The results shown are representative of at least 2 independent experiments.

The inhibition in Fpn1 surface expression induced by *L. amazonensis* infection was expected to increase the intracellular iron content of macrophages. To test this hypothesis, we used a ferrozine assay to quantify iron in BMDM infected or not with *L. amazonensis* amastigotes. The results showed a significant increase in the total iron content of wild type BMDM at 24 and 48 h after infection. This effect was not seen in TLR4^−/−^ BMDM (Table 1). At the same time points, infected wild type BMDM showed upregulation of the iron storage protein ferritin, at both the mRNA and protein levels (Figure 3A, B). This increase in intracellular iron content is likely to be a consequence of the parasite-induced inhibition of Fpn1 expression, which is expected to result in decreased iron export.

**Table 1:** Effect of *L. amazonensis* infection on the total iron content of macrophages.

		nmoles Fe/mg protein					
	Exp.	2 h		24 h		48 h	
		Control	Infected	Control	Infected	Control	Infected
WT	1	14.9	11.3	24.1	32.3	18	24.3
	2	19	13.3	16.6	22.3	11.6	26.2
	3	25.2	27.3	14.6	27.7	13.7	36.3
	Mean	19.7	17.3	18.4	27.4	14.4	28.9
TLR4 ^−^/^−^	1	16	14.8	11.3	12.1	16.3	12.2
	2	21.9	21.3	11.9	14.3	12.3	11.9
	Mean	18.9	18	11.6	13.2	14.3	12.05

WT and TLR4^−^/^−^ BMDM were infected or not with axenic *L. amazonensis* amastigotes (*L.a*) at a MOI = 5 for 1 h, washed to remove extracellular parasites, cultured at 34°C for another 2, 24 or 48 h, washed, frozen, lysed in 50 mM NaOH, and subjected to total protein and iron quantification assays. Results from independent experiments (Exp) are shown.

### Hepcidin-mediated downregulation of Fpn1 stimulates the intracellular growth of *L. amazonensis*

**Figure 4:**
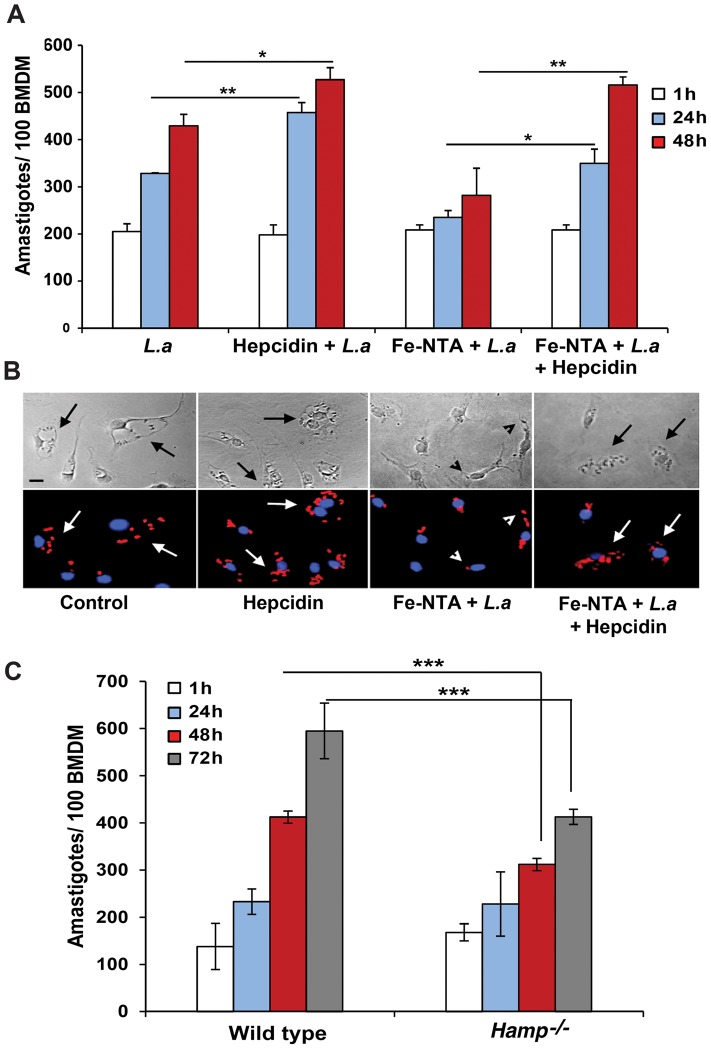
Hepcidin promotes *L. amazonensis* growth in macrophages. (**A**) Effect of iron loading and hepcidin on the intracellular replication of *L. amazonensis*. BMDM were infected with *L. amazonensis* axenic amastigotes (*L.a*). *L.a*, 1 h infection; *Hepcidin+L.a*, 4 h of hepcidin treatment followed by 1 h infection; *Fe-NTA+L.a*, overnight incubation with Fe-NTA followed by 1 h infection; *Fe-NTA+L.a+hepcidin*, overnight incubation with Fe-NTA followed by 1 h infection, parasite removal, and 18 h treatment with hepcidin. After washing, BMDM were cultured at 34°C for another 1, 24 or 48 h, fixed, stained and the number of intracellular parasites was determined microscopically. The values represent the mean +/− SD of 3 independent experiments. (*) p<0.05; (**) p<0.01 (unpaired Student's t test). (**B**) Representative images of BDMM infected as in (A), after 48 h Top panels, phase contrast; bottom panels, anti-*Leishmania* antibodies (red) and DAPI (blue) fluorescence. The arrows point to PVs containing replicating parasites; arrowheads point to single, non-replicating parasites. Bar  =  10 µm (applies to all images). (**C**) Intracellular growth of *L. amazonensis* in wild type or *Hamp* ^−/−^ macrophages. BMDM from WT and *Hamp* ^−/−^ mice were infected with WT axenic amastigotes of *L. amazonensis* for 1 h followed by washes, incubation at 34°C for 1, 24, 48 h or 72 h, staining with DAPI and quantification of the number of intracellular parasites. The values represent the mean +/− SD of triplicates (***) p<0.001 (unpaired Student's t-test).

We proceeded to investigate the impact of Fpn1-mediated iron export on the intracellular replication of *L. amazonensis*. In untreated BMDM the parasites increased in number over time, doubling in density by 48 h after infection. When BMDM were pre-treated with 1 µg/ml hepcidin for 4 h before infection, the intracellular growth rate of the parasites was enhanced (Figure 4A,B). These results suggest that hepcidin-mediated Fpn1 downregulation and block in iron export is beneficial for the establishment of *L. amazonensis* intracellular infections. After pre-treating BMDM with Fe-NTA, a condition that markedly stimulates Fpn1 expression (Figure 1), the opposite effect was observed: *L. amazonensis* intracellular replication was inhibited. This inhibitory effect of iron loading was apparently not due to iron toxicity, since Fe-NTA-treated BMDM remained >80% viable up to 48 h (Alamar Blue viability assay, results not shown). Furthermore, the inhibition in parasite replication was fully reversed by a subsequent exposure of the Fe-NTA-treated BMDM to hepcidin (Figure 4A,B). Microscopic examination showed that Fe-NTA-loaded macrophages contained small parasitophorous vacuoles (PV) with only one or two amastigotes. In contrast, after hepcidin treatment PVs were markedly expanded and housed numerous replicating parasites (Figure 4B). These findings suggest that even when macrophages are loaded with an iron source such as Fe-NTA, continuous iron efflux through Fpn1 on the plasma membrane has a negative impact on the ability of *L. amazonensis* to replicate in macrophages. Confirming the importance of hepcidin production by macrophages for the establishment of intracellular infections, the growth rate of *L. amazonensis* was reduced in BMDM prepared from *Hamp* ^−/−^ mice, when compared to their wild type counterparts (Figure 4C).

### Modulation of plasma membrane-associated Fpn1 controls the intracellular replication of *L. amazonensis*

**Figure 5:**
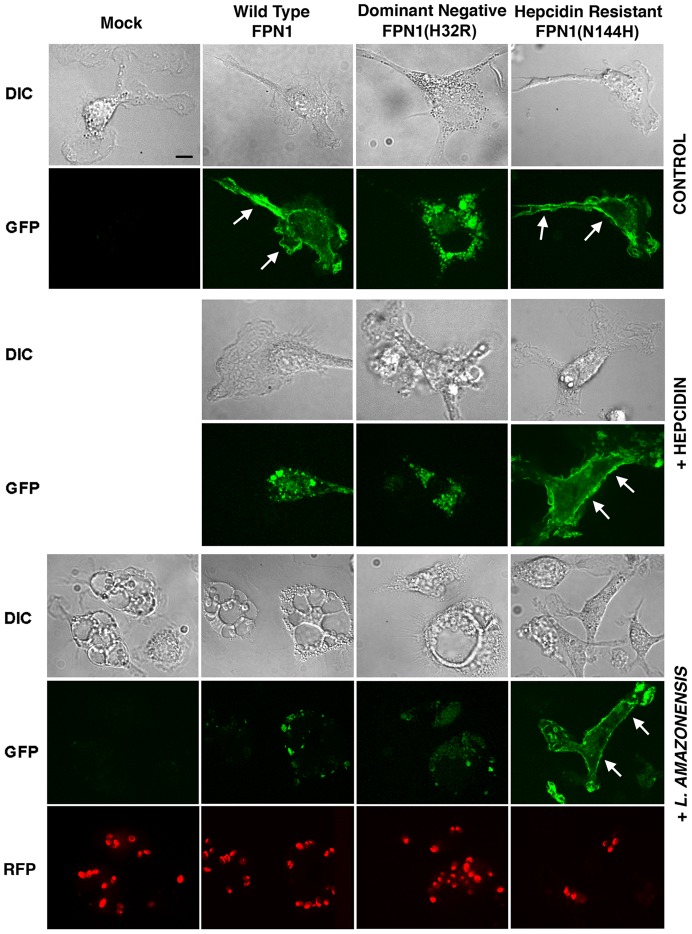
Fpn1 removal from the cell surface stimulates the intracellular growth of *L. amazonensis*. BMDM were mock transfected or transfected with Wild type Fpn1-GFP, Fpn1(H32R)-GFP or Fpn1(N144H)-GFP, and after 24 h were treated with hepcidin for 4 h or infected or not with axenic amastigotes of *L. amazonensis*-RFP for 1 h followed by washes and additional incubation at 34°C for 48 h. The images shown are representative of several live confocal images acquired in 2 independent experiments. The arrows point to Fpn1 on the plasma membrane of BMDM. Bar = 10 µm (applies to all images).

Our results suggested that hepcidin production during *L. amazonensis* infection reduces the amount of Fpn1 present on the surface of macrophages, a condition that promotes intracellular parasite replication. In order to directly visualize this process, we transfected BMDM with three different GFP-tagged constructs: Wild Type Fpn1, Fpn1(H32R) and Fpn1(N144H). Fpn1(H32R) has a dominant-negative effect, severely reducing the amount of Fpn1 that reaches the cell surface [13]. On the other hand, the N144H mutation renders Fpn1 resistant to hepcidin-mediated internalization and degradation, increasing levels at the cell surface [14]. As expected, we detected Fpn1-GFP on the plasma membrane of BMDM transfected with the wild type and hepcidin-resistant N144H constructs, and on intracellular compartments of BMDM expressing the dominant-negative H32R mutation (Figure 5, Control). Hepcidin treatment triggered removal of wild type Fpn1-GFP from the plasma membrane of macrophages, while the hepcidin-resistant Fpn1(N144H)-GFP version remained on the cell surface (Figure 5, +hepcidin). Confirming that *L. amazonensis* infection triggers removal of Fpn1 from the cell surface of BDMM, after 48 h of infection the level of wild type Fpn1-GFP associated with the plasma membrane was strongly reduced, with small amounts detected in intracellular compartments. In contrast, downregulation of surface-associated Fpn1 was not detectable in infected BMDM overexpressing the hepcidin-resistant Fpn1(N144H)-GFP (Figure 5, +*L. amazonensis*). These results further support the hypothesis that *L. amazonensis* downregulates Fpn1 expression by triggering endogenous hepcidin production by macrophages. The hepcidin-resistant version of Fpn1-GFP remained associated with the BMDM plasma membrane up to 72 h after infection (data not shown).

**Figure 6:**
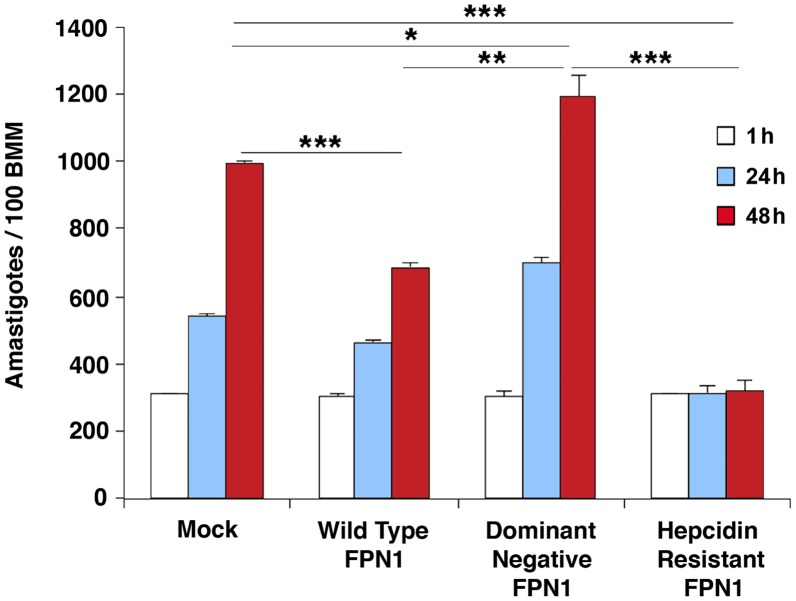
Modulation in plasma membrane-associated Fpn1 controls the intracellular growth of *L. amazonensis*. BMDM mock-transfected or transfected with wild type Fpn1-GFP, dominant-negative Fpn1(H32R)-GFP and hepcidin-resistant Fpn1(N144H) were infected with *L. amazonensis* for 1 h, washed and incubated at 34°C for 1, 24 or 48 h, followed by fixation, staining with DAPI and microscopic quantification of intracellular parasites. The values represent the mean +/− SD of triplicates, and the results are representative of 3 independent experiments. (*) p<0.05; (**) p<0.01; (***) p<0.001 (unpaired Student's t test).

Intracellular replication assays showed that *L. amazonensis* growth in BMDM overexpressing wild type Fpn1-GFP was inhibited, in agreement with the increased levels of surface-associated Fpn1 and lower levels of cellular iron. In contrast, expression of the dominant-negative Fpn1(H32R)-GFP construct that interferes with Fpn1 surface targeting stimulated parasite intracellular replication. Reinforcing the conclusion that intracellular growth of *L. amazonensis* depends on hepcidin-mediated removal of Fpn1 from the macrophage surface, parasite replication over a period of 48 h was strongly inhibited in BMDM expressing the hepcidin-resistant Fpn1(N144H)-GFP (Figure 6).

### Dominant-negative Fpn1 rescues the intracellular growth of *Leishmania* mutants defective in iron acquisition

**Figure 7:**
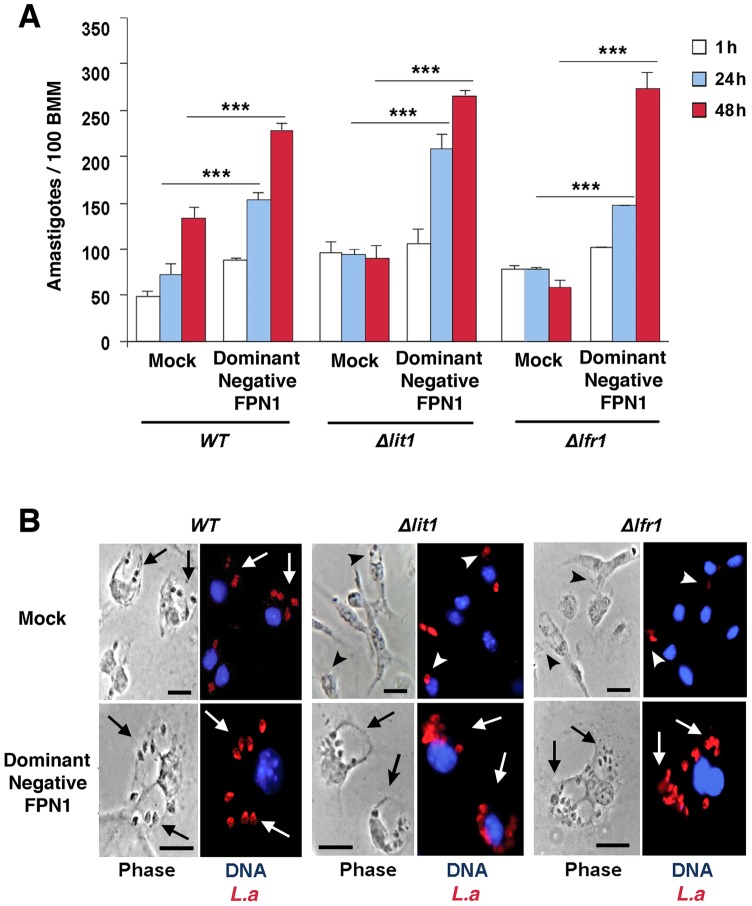
The intracellular growth of *L. amazonensis* mutants defective in iron uptake is rescued by dominant-negative Fpn1. (**A**) Effect of dominant-negative Fpn1 expression on the intracellular growth of *L. amazonensis*. BMDM were mock-transfected or transfected with Fpn1(H32R)-GFP, followed by infection with axenic amastigotes of wild type (WT), Δ*lit1* and Δ*lfr1 L. amazonensis* for 1 h, followed by washes, incubation at 34°C for 1, 24 or 48 h, fixation, staining and microscopic quantification of intracellular parasites. The values represent the mean +/− SD of triplicates, and the results are representative of 2 independent experiments. (***) p<0.001 (unpaired Student's t test). (**B**) Representative images of BMDM infected for 48 h as described in (A). Left panels, phase contrast; right panels, anti-*Leishmania* antibodies (red), DAPI (blue) fluorescence. Arrows point to replicating parasites; arrowheads point to single, non-replicating parasites. Bars = 10 µm.

**Figure 8:**
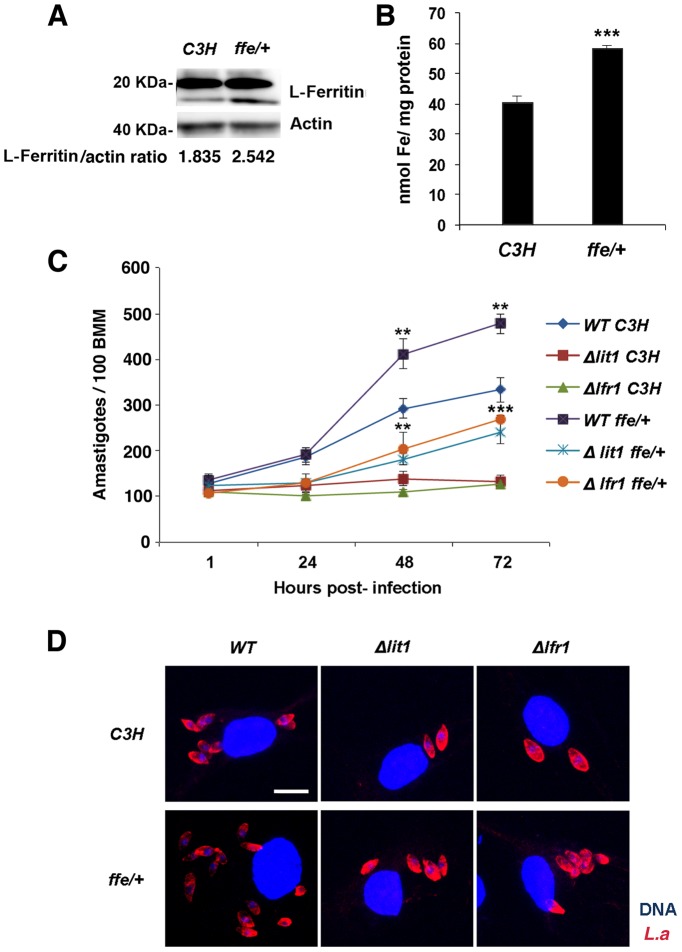
Macrophages from flatiron mice, a model for hereditary hemochromatosis type IV, are more susceptible to infection by *L. amazonensis*. **A**) L-ferritin protein levels in wild-type (*C3H*) and flatiron (*ffe/+*) macrophages. BMDM from *C3H* or *ffe/+* mice were solubilized and analysed by SDS-PAGE and Western Blot with anti-L-ferritin antibodies followed by peroxidase-conjugated secondary antibodies. Anti-actin antibodies were used as loading controls. L-ferritin/actin ratios were determined by quantitative digital imaging. **B**) Total iron levels in wild-type (*C3H*) and flatiron (*ffe/+*) macrophages. BMDM from *C3H* or *ffe/+* mice were washed, frozen, lysed in 50 mM NaOH, and subjected to total protein and ferrozine iron quantification assays. The values represent the mean +/− SD of triplicates. (***) p<0.001 (unpaired Student's t-test). (**C**) Intracellular growth of *L. amazonensis* in *C3H* and *ffe/+* macrophages. BMDM from *C3H* and *ffe/+* mice were infected with *WT*, *Δlit1* and *Δlfr1* axenic amastigotes of *L. amazonensis* for 1 h followed by washes, incubation at 34°C for 1, 24 or 48 h, staining and quantification of the number of intracellular parasites. The values represent the mean +/− SD of triplicates, and the results are representative of 2 independent experiments. (***) p<0.001 (unpaired Student's t-test). (**D**) Representative confocal fluorescence images of BMDMs infected for 72 h with the *L. amazonensis* (*L.a.*)strains as described in (C). Red, anti-*Leishmania* antibodies; Blue, DAPI.Bar = 10 µm (applies to all images).

We hypothesized that the enhanced replication of wild type *L. amazonensis* in BMDM expressing dominant-negative Fpn1(H32R) was due to an increased availability of intracellular iron to the parasites. To test this hypothesis, we examined the effect of dominant-negative Fpn1 on the intracellular fate of *L. amazonensis* null mutant strains deficient in iron uptake. Strains lacking the LIT1 ferrous iron transporter (Δ*lit1*) and the LFR1 ferric iron reductase (Δ*lfr1*) are incapable of growing inside macrophages, but their replication can be fully rescued by loading the endosomal compartment of host macrophages with an abundant iron source, cationic ferritin [6]. Strikingly, we found that expression of dominant-negative Fpn1 restored intracellular growth rates of Δ*lit1* and Δ*lfr1* null strains to levels comparable to wild type *L. amazonensis* (Figure 7A, B). A similar effect was observed in BMDM from flatiron mice [13], which carry the dominant-negative H32R mutation in one *Fpn1* allele, and have elevated levels of ferritin (Figure 8A) and iron (Figure 8B). The Δ*lit1* and Δ*lfr1* null parasite strains did not grow intracellularly in control C3H BMDM, but were able to replicate in *ffe/+* BMDM (Figure 8C,D). Collectively, these results show that Fpn1 removal from the cell surface facilitates *L. amazonensis* access to iron while replicating inside macrophage PVs, and compensates for specific defects in the parasite's iron acquisition pathway.

## Discussion

**Figure 9:**
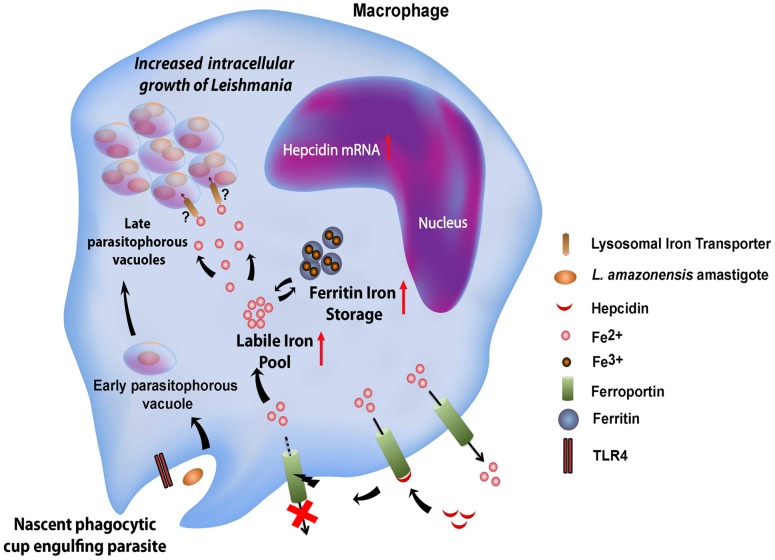
Proposed model for the *L. amazonensis*-induced downregulation of ferroportin expression in macrophages. Macrophages infected by *Leishmania amazonensis* produce hepcidin, a peptide hormone that binds to the iron exporter ferroportin, inducing its internalization and degradation. This causes an increase in the macrophage cytosolic iron content, which includes the bioavailable labile iron pool and iron stored as a complex with ferritin. By an unknown mechanism, Fpn1 internalization and elevated cytosolic iron increases iron availability inside *Leishmania*-containing parasitophorous vacuoles, stimulating parasite growth.

Genes involved in iron acquisition play a critical role in pathogen virulence, as extensively demonstrated in bacteria [15] and more recently in the protozoan parasite *Leishmania amazonensis* [6], [7]. In mammals *Leishmania* is an obligate intracellular parasite of macrophages, replicating within PVs with properties of phagolysosomes. Utilization of the macrophage as host cell has important implications for how *Leishmania* gains access to iron. Macrophages from the reticuloendothelial system play a fundamental role in iron recycling in vivo, through the process of erythrophagocytosis. Heme released from phagocytosed senescent red blood cells is translocated to the macrophage cytosol, where iron is extracted through the activity of heme oxygenase. Cytosolic iron is then utilized by the cell, stored as a complex with ferritin, or exported by Fpn1 [1]. In this study we showed that *L. amazonensis* directly interferes with the iron export function of macrophages, by inhibiting cell surface expression of Fpn1. This *Leishmania*-driven process is associated with increased total macrophage iron content and stimulation of parasite intracellular replication (Figure 9).

Consistent with an inhibitory effect on iron export, infection by *L. amazonensis* enhanced expression of ferritin, the cytosolic iron storage protein. At the protein level, this observation is consistent with the presence of a 5′ IRE in the ferritin mRNA [16]. However, the total amount of ferritin mRNA was also elevated after *L. amazonensis* infection. This indicates that signaling events regulating transcription, possibly associated with macrophage inflammatory responses, are also triggered during infection and participate in the cellular response to elevated iron pools. Indeed, we found that the ability of *Leishmania* to inhibit Fpn1 expression depends on TLR4, a major pattern-recognition receptor of macrophages that is commonly associated with pro-inflammatory responses. Different species of *Leishmania* were described to elicit distinct TLR4-mediated responses in macrophages, although the exact identity of the molecule(s) serving as TLR4 ligand(s) remain unknown [17]. While TLR4 plays a protective role in infections with *L. major* [18], it appears to have an anti-inflammatory effect in macrophages infected with *L. mexicana*, a species closely related to the *L. amazonensis* used in our study [19]. We found that TLR4-dependent downregulation of Fpn1 requires live parasites, suggesting that the molecule(s) involved may be actively secreted, or may only come in contact with the TLR4 receptors in an intracellular compartment generated during cell invasion.

Several lines of evidence indicate that *L. amazonensis*-induced Fpn1 downregulation is mediated, at least in part, by production of the peptide hormone hepcidin by infected macrophages. First, there was a marked increase in hepcidin transcripts in infected macrophages. Second, Fpn1 carrying the N144H mutation that renders it resistant to hepcidin-mediated internalization and degradation [13] remained on the plasma membrane of infected macrophages, while wild type Fpn1 was removed. Third, the effects of *L. amazonensis* infection on both hepcidin and Fpn1 expression in macrophages were TLR4-dependent. Hepcidin is produced in the liver during inflammation and other stimuli in a paracrine manner [20], but it can also be produced locally by macrophages in an autocrine fashion in response to infections, as previously shown for *Pseudomonas aeruginosa* [21] and *Mycobacterium tuberculosis* [22]. Similar to our findings with *L. amazonensis*, hepcidin production and decrease cell surface-associated Fpn1 triggered by infection with *Pseudomonas aeruginosa* require TLR4 [21].

Earlier studies demonstrated that Fpn1 containing the H32R mutation has a dominant-negative effect, preventing the traffic of Fpn1 to the cell surface [13]. We found that Fpn1 carrying this mutation enhances *L. amazonensis* intracellular replication when expressed in macrophages. This was observed after expression of Fpn1(H32R)-GFP, and also in macrophages from flatiron mice, which are heterozygous for this mutation and represent a model for the autosomal-dominant human type IV hereditary hemochromatosis type A [13]. Notably, macrophages expressing Fpn1(H32R)-GFP or isolated from flatiron mice rescued the intracellular growth defect of *LFR1* and *LIT1* null mutants deficient in iron uptake [6], [7], directly supporting the conclusion that Fpn1 removal from the cell surface facilitates parasite access to iron.

Our findings indicate that blocking Fpn1 surface expression increases iron availability in the cytosol, which is associated with *L. amazonensis* intracellular replication. This result suggests the existence of a crosstalk between the cytosolic iron pool and the iron content of L. amazonensis PVs, a process that is still poorly understood (Figure 9). Interestingly, we found that increasing ferritin-iron cytosolic stores by treating macrophages with an exogenous iron source is not sufficient to promote parasite replication. Hepcidin-mediated downregulation of Fpn1 appears to be required to facilitate amastigote access to iron, suggesting that Fpn1 internalization promotes formation of a cytosolic iron pool that can be more readily mobilized by the parasites. Evidence for such a mechanism was recently obtained in breast cancer cells, where hepcidin-mediated internalization of Fpn1 was associated with an increased labile iron pool [23]. Another intriguing possibility is that Fpn1 may be delivered to the membrane of the lysosome-like PV after hepcidin-mediated internalization, where it may remain transiently active and transport cytosolic iron into the lumen of the parasite-containing vacuole.

Fpn1-mediated iron depletion inhibits the intracellular growth of several bacterial pathogens, such as *Chlamydia psittaci*, *Chlamydia trachomatis*, *Legionella pneumophila* [24]. Elevated Fpn1 expression was also identified as a host defense mechanism against infections with *Salmonella typhimurium* and *Mycobacterium tuberculosis* [25]. To our knowledge, the present study represents the first demonstration that a protozoan pathogen blocks Fpn1 expression as a strategy to facilitate their intracellular replication. This strategy may complement additional mechanisms that facilitate parasite access to iron, such as the elevated expression of transferrin receptors observed in macrophages infected with *Leishmania donovani* [26]. Our study demonstrates the important role played by cytosolic iron pools in *L. amazonensis* infections, and opens the way for future investigations of how iron enters the PV, and whether differential access to iron in cutaneous or visceral sites influences the clinical disease caused by different *Leishmania* species.

## Materials and Methods

### Ethics statement

All animal work was conducted according to the National Institutes of Health guidelines for the housing and care of laboratory animals and performed under protocol # R-11-73 approved by the University of Maryland College Park Institutional Animal Care and Use Committee on January 3, 2013. The University of Maryland at College Park is a fully AAALAC-accredited institution.

### *Leishmania* parasite

Wild type *Leishmania amazonensis* (IFLA/BR/67/PH8 strain, expressing or not RFP) or knockout strains (Δ*lfr1* and Δ*lit1*; Flannery et al., 2011; Huynh et al., 2006) promastigotes were maintained *in vitro* at 26°C in M199 (Invitrogen), 40 mm HEPES, pH 7.4, 20% heat-inactivated FBS, 5% penicillin/streptomycin, 0.1% hemin (from a 25 mg/ml stock in 50% triethanolamine), 10 mm adenine, and 5 mm l-glutamine. To obtain axenic amastigotes, stationary phase cultures rich in metacyclic promastigotes were incubated at 32°C in acidified media, as previously described [6]. Heat-killed (65°C incubation for 1 h) axenic amastigotes were washed, resuspended in BMDM medium and added to BMDM at a MOI = 5.

### Mouse Bone Marrow Macrophages (BMDM)

BMDM obtained from WT or TLR4^−^/^−^ C57Bl/6 mice (The Jackson Laboratory), C3H mice or Flatiron (ffe/+ heterozygous) mice in the C3H genetic background (provided by D. Ward and J. Kaplan, U. Utah) or *Hamp* ^−/−^ C57BL/6 mice (provided by S. Vaulont, Cochin Institite - [27]) were prepared as described [28] and cultured in RPMI 1640 containing 10% heat-inactivated FBS, 100 units/ml penicillin and 100 µg/ml streptomycin and 50 ng/ml human Recombinant M-CSF (Peprotech). Mature, adherent BMDM were detached from dishes with PBS/1 mM EDTA, viability was determined with a trypan-blue exclusion test, cells were seeded into tissue culture-treated 6 well plates at a density of 2×10^6^ cells/well, and incubated overnight at 37°C in a 5% CO2 incubator prior to use in experiments.

### BMDM treatment and infection

BMDM were plated on glass coverslips in RPMI 10% FBS. After 24 h, BMDM were incubated with freshly prepared Fe-nitrilotriacetate (Fe-NTA, 1∶4 solution of FeCl3 200 µM and NTA 400 µM) for 16 h, and washed before infection. For infection, axenic amastigotes were added to BMDM for 1 h at 34°C at a MOI = 5, washed in PBS to remove non-internalized parasites, reincubated for 1, 24, 48 or 72 h at 34°C, fixed with 4% PFA, stained with DAPI and anti-*L. amazonensis* polyclonal mouse antibodies, and mounted (Prolong Antifade; Molecular Probes). Intracellular parasites were quantified microscopically, as previously described [29]. To assess the effect of hepcidin on inhibition of ferroportin expression and parasite growth, BMDM were treated for 4 or 18 h with 1 µg/ml hepcidin (kindly provided by J. Kaplan and D. M. Ward, University of Utah) [29].

### Plasmids and BMDM transfection

Fpn1-GFP constructs were kindly provided by J. Kaplan and D. M. Ward, University of Utah) [13]. BMDM were transfected with wild type ferroportin (Fpn1 -GFP), hepcidin-resistant ferroportin (Fpn1(N144H)-GFP) and dominant-negative Ferroportin (Fpn1(H32R)-GFP). 4 µg of each plasmid was used to transfect 1×10^6^ BMDM using nucleofactor technology (Amaxa) according to the manufacturer's instructions. As controls, BMDMs were mock-transfected with the plasmid elution buffer.

### BMDM viability assay

1×10^6^ BMDM plated in 96 well plates were treated with Fe-NTA (150, 200, 400, 800 µM) or 0.1% saponin for 24 h, washed in PBS, and incubated in medium containing 10% Alamar Blue Cell Viability Reagent (Invitrogen) at 37°C for 4 h protected from light. The fluorescence signal generated by the fluorimetric redox indicator was detected using a SpectraMAX M5 microplate fluorimeter at 560 nm excitation and 590 nm emission, and analyzed using software SoftMAX R _ PRO (Molecular Devices).

### Imaging

For live imaging, transfected BMDM were plated in 35 mm glass-bottom dishes (Mattek), infected or not with axenic amastigotes for 1 h, washed and reincubated for 48 h. Dishes were then transferred to an environmental chamber at 34°C with 5% CO_2_ and humidity control (Pathology Devices). Confocal stacks were acquired using a spinning disk UltraVIEW VoX system (PerkinElmer) attached to an Eclipse Ti inverted microscope (Nikon) with a 60×1.4 N.A. objective and equipped with a C9100-50 camera (Hamamatsu), or on a Leica TCS SP5 X Supercontinuum confocal microscope using a 63× NA 1.2 objective. Acquisition was performed using Volocity Suite (PerkinElmer), or Leica application suite software package (Leica).

### Quantitative real time PCR

C57BL/6 BMDMs plated at 2×10^6^ in 6 well plates were loaded or not with Fe-NTA, and infected or not with *L. amazonensis* axenic amastigotes for 1 h. BMDM were washed in PBS and reincubated for 1, 24 or 72 h before lysis and RNA extraction (Nucleospin RNA II kit, Macherey-Nagel) according to the manufacturer's instructions. For in *vivo* analysis of hepcidin mRNA levels, five week-old female C57BL/6 mice were inoculated with 1×10^6^ purified metacyclics of wild type *L. amazonensis* (Pinto-da-Silva et al., 2005) in the left hind footpad in a volume of 50 µl PBS. After 9 weeks, liver or footpad from 3 infected mice were recovered and vigorously homogenized using micro tissue grinder (Radnoti LLC). Non-infected mice were used as experiment controls. The samples were kept on ice for subsequent RNA extraction. cDNA synthesis was carried out using 1 µg of total RNA and Superscript II Reverse Transcriptase (Invitrogen) according to the manufacturer's protocol. To quantify transcript levels, 1 µl of cDNA was amplified using the following primers: mouse FPN1-Forward 5′- CTA CCATTAGAAGGATTGACCAGCT-3′, mouse FPN1-Reverse 5′- ACTGGAGAACCAAATGTCATAATCTG-3′; mouse HAMP- Forward 5′-TGTCTCCTGCTTCTCCTCCT-3′, mouse HAMP-Reverse5′- CTCTGTAGTCTGTCTCATCTGTTG-3′; mouse FTH1-Forward 5′- GCGAGGTGGCCGAATCT-3′, mouse FTH1-Reverse 5′- CAGCCCGCTCTCCCAGT-3′; mouse HPRT1-Forward 5′- TCAGTCAACGGGGGAACATAAA-3′, mouse HPRT1-Reverse 5′- GGGGCTGTACTGCTTAACCAG-3′, mouse β-III-Tubulin-Forward 5′-CGCACGACATCTAGGACTGA-3′, mouse β-III-Tubulin-Reverse 5′- TGAGGCCTCCTCTCACAAGT-3′ Real-time PCR was performed using a BioRad iQ icycler Detection system (BioRad Laboratories) using SYBR green fluorophore (BioRad Laboratories) according to the manufacturer's instructions. Fluorescence was detected at each annealing step, and the cycle threshold (*C*_t_) was calculated by determining the point at which the fluorescence exceeded a threshold limit. All reactions were performed in triplicate, and negative controls (no template cDNA) were included in each experiment. Data were normalized to the level of HPRT1 (mouse BMDM) or tubulin (mouse liver and footpad) expression in each sample.

### Protein extraction and western blot

BMDM were washed twice with cold PBS, scraped from the dish, collected by centrifugation (335 *g*) for 10 min at 4°C, lysed in 30 µl RIPA buffer (50 mM Tris-HCl, pH 8.0, 150 mM NaCl, 1% Nonidet P-40, 0.50% sodium deoxycholate, 0.10% SDS) with protease inhibitors (Roche), and centrifuged at 14000 g for 15 min at 4°C to remove insoluble material. Lysates were assayed for protein content (Pierce BSA Protein Assay kit), 60 µg per sample were mixed with SDS sample buffer at room temperature for 30 min, separated by SDS-PAGE (without sample boiling) and transferred to a PVDF membrane (Millipore). Membranes were incubated overnight with 1∶1000 rabbit anti-Fpn1 antibody (Alpha Diagnostics), 1∶800 anti L-ferritin (Abcam) or 1∶5000 anti-actin (Sigma) in blocking buffer (PBS 3% milk, 0.1% Tween), followed by HRP-coupled anti-rabbit IgG or anti mouse IgG secondary antibodies (Amersham Biosciences). Blots were developed using Immun-Star HRP Luminol Enhancer and peroxidase buffer (Bio-Rad) and detected with a Fuji LAS-3000 Imaging System and Image Reader LAS-3000 software. Digital quantifications of chemiluminescence were performed using Image J software.

### Intracellular iron quantification

A ferrozine-based colorimetric assay was performed as previously described [30]. Briefly, BMDMs were infected or not with *L. amazonensis* axenic amastigotes for 1 h and reincubated for 2, 24 or 48 h. At different time points, the dishes were washed twice with ice-cold PBS and stored at −20°C. Cells were lysed with 200 µl of 50 mM NaOH for 2 h on a shaker in a humidified atmosphere and aliquots were used for iron and protein determinations. For iron quantification, 100 µl of lysates were mixed with 100 µl 10 mM HCl, and 100 µl of the iron-releasing reagent (a freshly mixed solution of equal volumes of 1.4 M HCl and 4.5% (w/v) KMnO_4_). After for 2 h at 60°C in a fume hood, samples were cooled to room temperature and 30 µl of the iron-detection reagent (6.5 mM ferrozine, 6.5 mM neocuproine, 2.5 M ammonium acetate, and 1 M ascorbic acid) was added to each sample. After 30 min, 280 µl of each sample was transferred into a 96-well plate and the absorbance measured at 550 nm in a microplate reader. The iron content in each sample was calculated based on a standard curve (0–300 µM FeCl_3_) prepared under the same conditions, and normalized to protein levels. Protein levels were determined by a modified Lowry assay (Pierce) per manufacturer's instructions.

## Supporting Information

Supporting Information S1 **Raw data of qPCR assays detecting hepcidin (** ***Hamp*** **) in mouse tissues.** The tables provided show the Ct values obtained in real time PCR assays with RNA isolated from 3 uninfected mice or 3 mice infected with *L. amazonensis* using specific primers for *Hamp* or tubulin, as described in Materials and Methods. The table also indicates how normalization was done using the values for tubulin transcripts.
(PDF)
