# Supplementary material for: Leishmania-Mediated Inhibition of Iron Export Promotes Parasite Replication in Macrophages
Source: PLoS Pathog. 2014 Jan 30;10(1):e1003901. doi: 10.1371/journal.ppat.1003901 (PMC3907422; doi:10.1371/journal.ppat.1003901)
Supplement: Supporting Information S1 — Raw data of qPCR assays detecting hepcidin ( Hamp ) in mouse tissues. The tables provided show the Ct values obtained in real time PCR assays with RNA isolated from 3 uninfected mice or 3 mice infected with L. amazonensis using specific primers for Hamp or tubulin, as described in Materials and Methods. The table also indicates how normalization was done using the values for tubulin transcripts. (PDF) [file ppat.1003901.s001.pdf]

QPCR HAMP DATA NORMALIZATION

	C_t Values		(1/2^C_t)/(1/2^45)		Avg. triplicate	Hamp Values		
	Hamp	Tubulin	Hamp	Tubulin	Tubulin	normalized to Tubulin	Average	SD
Liver Non infected mouse 1	18.25	28.51	112863206.3	92041.69938	96218.66352	1172.986635		
	19.02	28.41	66184956.44	98647.85084		687.8598602	953.65861	245.87933
	18.48	28.42	96231107.72	97966.44036		1000.129332		
Liver Non infected mouse 2								
	17.08	26.1	253955415.8	489178.0011	477071.7348	532.3212365		
	17.04	26.12	261095074.3	482443.343		547.2868235	546.13151	13.270395
	17.01	26.19	266581236.9	459593.8604		558.7864831		
Liver Non infected mouse 3								
	17.58	27.68	179573596.6	163621.1817	178618.2969	1005.348275		
	17.55	27.37	183346819.6	202842.4388		1026.472779	849.54243	288.35151
	18.54	27.63	92311048.8	169391.2702		516.8062309		
Liver Infected mouse 1								
	18.42	28.18	100317634.9	115697.6471	80803.40129	1241.502626		
	18.22	29.24	115234702.2	55492.30079		1426.112025	1286.1812	123.79379
	18.48	28.88	96231107.72	71220.25595		1190.928924		
Liver Infected mouse 2								
	17.97	28.47	137037927.8	94629.34376	98461.52136	1391.791696		
	18.3	28.4	109018671.2	99334.0009		1107.221072	1249.5064	142.28531
	18.19	28.37	117656028.3	101421.2194		1249.506384		
Liver Infected mouse 3								
	17.45	28.57	196506255.7	88292.29969	90939.36422	2160.849236		
	17.78	28.41	156327895.7	98647.85084		1719.034403	1916.0061	224.76398
	17.66	28.61	169886974.2	85877.94212		1868.134616		
Footpad Non infected mouse 1								
	27.64	25.03	168221.1992	1026996.618	1182462.81	0.142263416		
	27.73	24.71	158047.6436	1282031.252		0.133659716	0.1264489	0.0203993
	28.1	24.76	122294.5003	1238360.561		0.103423549		
Footpad Non infected mouse 2								
	26.58	24.54	350729.6809	1442360.137	1437224.745	0.244032593		
	26.32	24.41	419991.324	1578365.613		0.292223833	0.2439145	0.0483684
	26.9	24.7	280958.9826	1290948.484		0.195487159		
Footpad Non infected mouse 3								
	27.52	25.25	182811.8418	881743.7995	873873.8244	0.209197068		
	27.18	25.23	231395.2943	894052.4843		0.264792568	0.2397811	0.0282136
	27.29	25.31	214408.1172	845825.1893		0.245353633		
Footpad Infected mouse 1								
	32.57	32.79	5518.268731	4737.794801	3488.131394	1.582012862		
	32.7	33.42	5042.767517	3061.451261		1.445693108	2.0935615	1.0063954
	31.53	33.62	11346.81676	2665.14812		3.252978595		
Footpad Infected mouse 2								
	33.02	33.14	4039.609158	3717.19846	3217.518486	1.255504568		
	33.23	33.22	3492.392517	3516.684028		1.085430443	1.1322281	0.1077871
	33.27	33.76	3396.89286	2418.672971		1.055749291		
Footpad Infected mouse 3								
	31.03	30.79	16046.82216	18951.17921	15993.45574	1.003336766		
	31.05	31.2	15825.90081	14263.10043		0.989523532	0.963624	0.0572402
	31.19	31.15	14362.30814	14766.08758		0.89801156		

	Avg Hamp Values normalized to Tubulin
Liver Non infected mouse 1	953.6586092
Liver Non infected mouse 2	546.1315144
Liver Non infected mouse 3	849.5424282
Liver Infected mouse 1	1286.181192
Liver Infected mouse 2	1249.506384
Liver Infected mouse 3	1916.006085
Footpad Non infected mouse 1	0.126448894
Footpad Non infected mouse 2	0.243914528
Footpad Non infected mouse 3	0.23978109
Footpad Infected mouse 1	2.093561522
Footpad Infected mouse 2	1.132228101
Footpad Infected mouse 3	0.963623953

Avg of normalized Hamp values from 3 individual mice

	Avg	SD
**Liver Non Infected**	783.1108506	211.7296605
**Liver Infected**	1483.897887	374.6656932

	Avg	SD
**Footpad Non infected**	0.203381504	0.066657642
**Footpad infected**	1.396471192	0.609555605
